# Descriptive statistics and visualization of data from the *R* datasets package with implications for clusterability

**DOI:** 10.1016/j.dib.2019.104004

**Published:** 2019-05-24

**Authors:** Naomi C. Brownstein, Andreas Adolfsson, Margareta Ackerman

**Affiliations:** a Department of Biostatistics and Bioinformatics, Moffitt Cancer Center, 12902 USF Magnolia Drive, Tampa, FL, 32612, USA; b Department of Behavioral Sciences and Social Medicine, Florida State University, 1115 West Call Street, Tallahassee, FL, 32306-4300, USA; c Department of Computer Engineering, Santa Clara University, 500 El Camino Real, Santa Clara, CA, 95053, USA

**Keywords:** Datasets, Dimension reduction, Histograms, Principal component analysis, Pairwise distances

## Abstract

The manuscript describes and visualizes datasets from the *datasets* package in the *R* statistical software, focusing on descriptive statistics and visualizations that provide insights into the clusterability of these datasets. These publicly available datasets are contained in the *R* software system, and can be downloaded at https://www.r-project.org/, with documentation provided at https://stat.ethz.ch/R-manual/R-devel/library/datasets/html/00Index.html. Further information on clusterability is found in the companion to this article, *To Cluster or Not to Cluster: An Analysis of Clusterability Methods*? (https://doi.org/10.1016/j.patcog.2018.10.026).

Brief descriptions and graphs of the variables contained in each dataset are provided in the form of means, extrema, quartiles, standard deviation and standard error. Two-dimensional plots for each pair of variables are provided. Original references to the data sets are included when available. Further, each dataset is reduced to a single dimension by each of two different methods: pairwise distances and principal component analysis. For the latter, only the first component is used. Histograms of the reduced data are included for every dataset using both methods.

**Table:** Specifications Table

Subject area	Machine Learning/Statistics
More specific subject area Clustering and clusterability	
Type of data	Plots and histograms
How data was acquired	Raw data publicly available
Data format	raw
Experimental factors	Data includes multiple component data sets downloaded from the R datasets package [13]. Data collection details for each component dataset are included Section 2.
Experimental features	Histograms of distances and first principal component of each dataset plus descriptive statistics and 2D plots of the raw data
Data source location	Data consists of multiple component data sets, collected from multiple locations, ranging from the United States to Switzerland, as described in Section 2.
Data accessibility	Data is in the *R* datasets package [13], available within *R* and at https://stat.ethz.ch/R-manual/R-devel/library/datasets/html/00Index.html
Related research article	Adolfsson A, Ackerman M, and Brownstein NC, “To Cluster, or Not to Cluster: An Analysis of Clusterability Methods.” *Pattern Recognition*, 88, 13–26 [1]. https://doi.org/10.1016/j.patcog.2018.10.026

**Table:** 

**Value of the Data** • *R* is a free, powerful statistical programming language compatible with Windows and Unix systems and is utilized by statisticians, computer scientists, data scientists, and other analysts, such as biologists working in genomics. The *R* datasets package [12], [13] includes dozens of datasets for use in education and research. Data are often used for demonstration by students learning established methodology and researchers testing new methods. • Descriptive statistics for all features and visual presentation of the data in the form of two-dimensional plots in a single document may help researchers and students to quickly comprehend the content of the data and evaluate which data may be best suited to their goals. • Simultaneous presentation of the descriptive statistics and each trio of plots (original 2D plots of data, distances and principal component) show researchers the component features and their ranges in each dimension and two unidimensional visual summaries for each dataset. • The first principal component is a useful one-dimensional summary for each dataset. Histograms of pairwise distances yield one-dimensional visualizations applicable to cluster analysis. These visual summaries are easier for researchers to evaluate for research and educational purposes than raw data or text. • The graphs presented have implications for clustering and clusterability, as described in our accompanying article [1].

## 1 Data

This paper highlights statistical summaries and visualizations with nine tables and eighteen figures for selected data from the *datasets* package within *R* software [12], [13], detailed in Section 2. Tables provide means, medians, ranges, standard deviations, and standard errors for all variables. Figures highlight plots of each pair of variables and unidimensional summaries of all datasets. For Fig. 10, Fig. 11, Fig. 12, Fig. 13, Fig. 14, Fig. 15, Fig. 16, Fig. 17, Fig. 18, the left plots are histograms of the sets of pairwise Euclidian distances for the corresponding dataset, and the right are histograms of the first principal components (PC1).

**Fig. 1:**
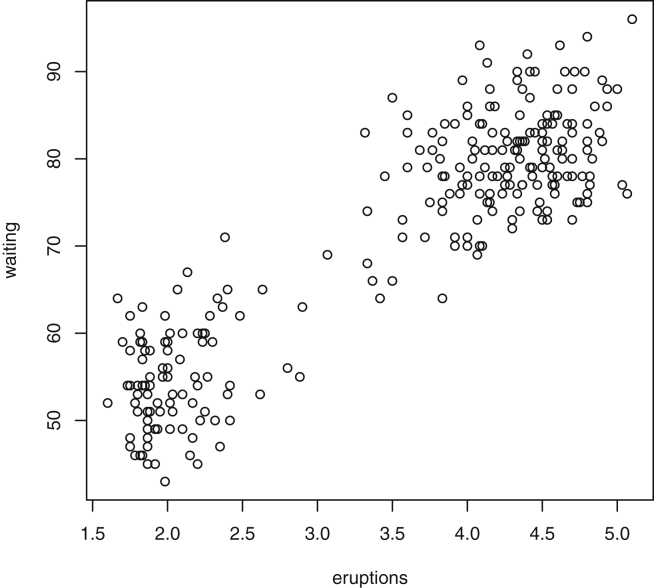
Plot of *Faithful* Data. Waiting time between eruptions vs. eruption duration, both measured in minutes.

**Fig. 2:**
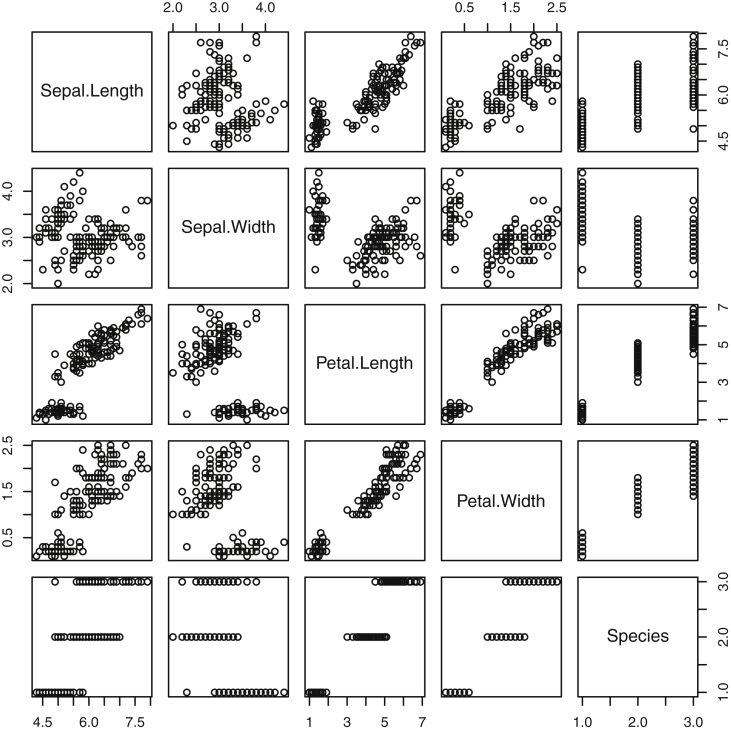
This figure shows 2D projections of the famous *iris* dataset. The first four variables are measured in centimeters. The fifth variable is an indicator of which of three species the observation belongs.

**Fig. 3:**
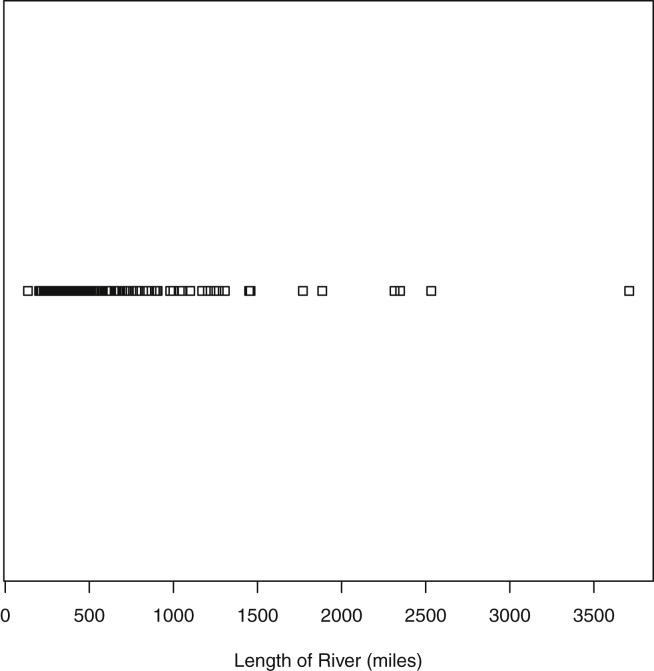
Plot of Rivers Data. Lengths are measured in miles.

**Fig. 4:**
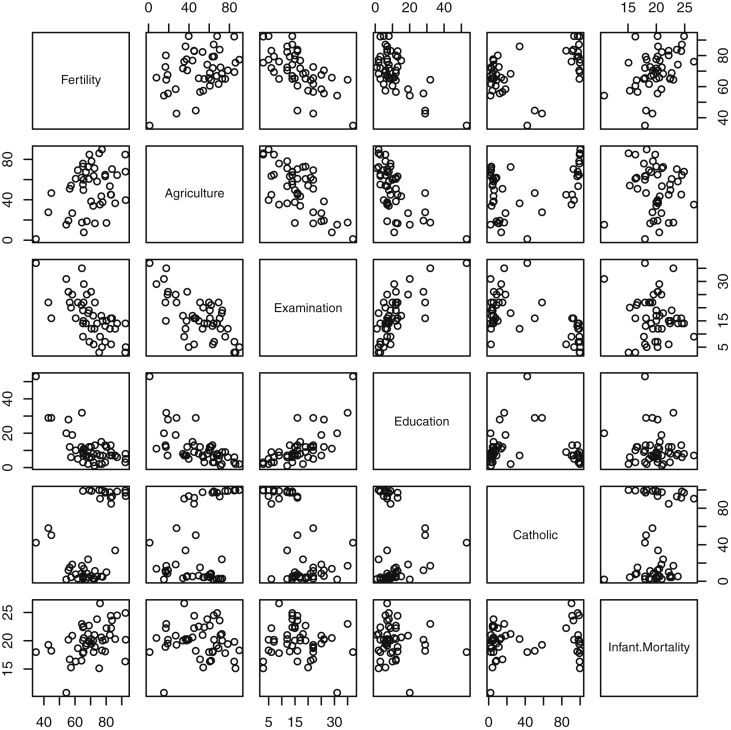
Pairwise plot of variables in the *swiss* data. Fertility is a standardized measure. All other variables are proportions of the populations falling into a certain category: agricultural job, high performance on the army exam, educational attainment past primary school, Catholic religion membership, and infant mortality.

**Fig. 5:**
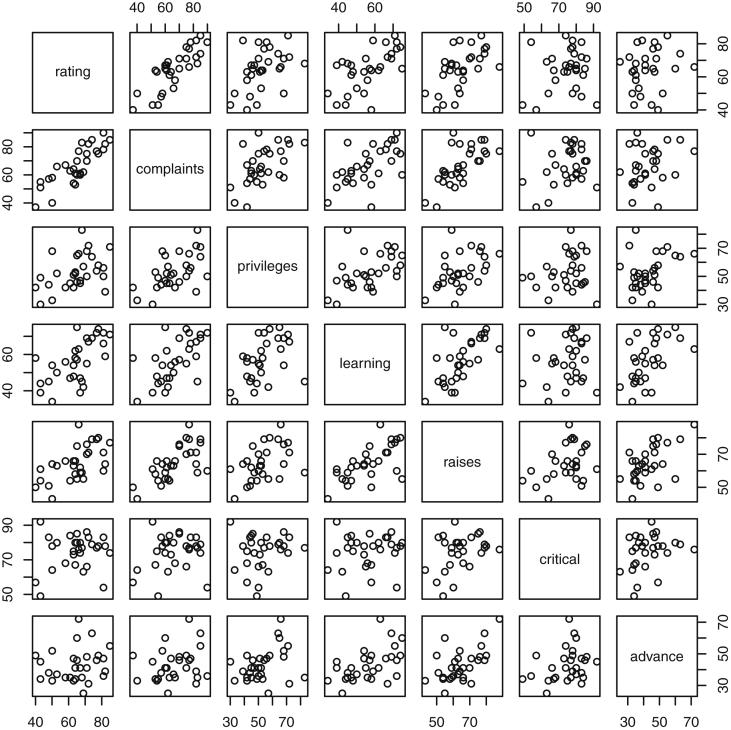
Plots of Attitude Data. Responses correspond to the percentage of favorable responses within a department on the corresponding topic.

**Fig. 6:**
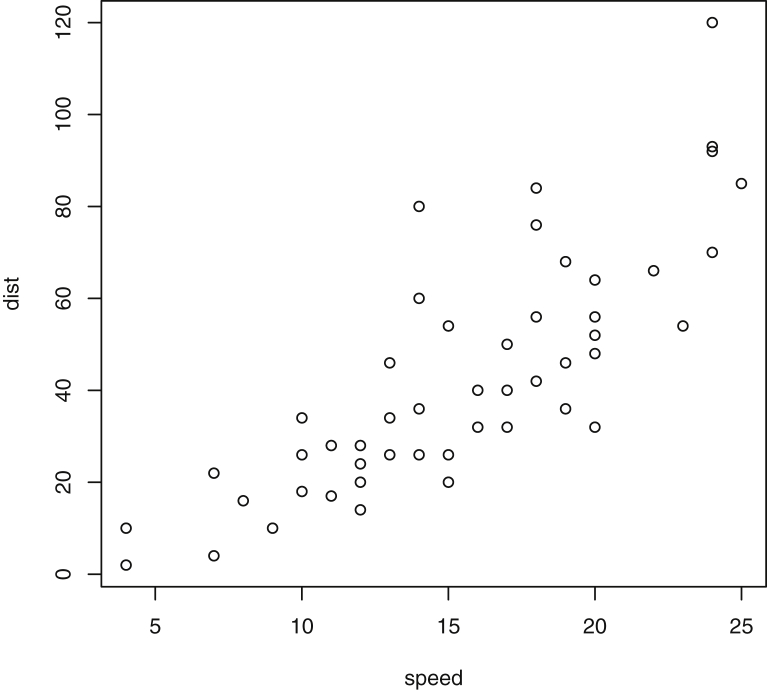
Plot of Cars Data: Stopping distance vs. speed. Stopping distance was measured in feet, and speed was measured in miles per hour.

**Fig. 7:**
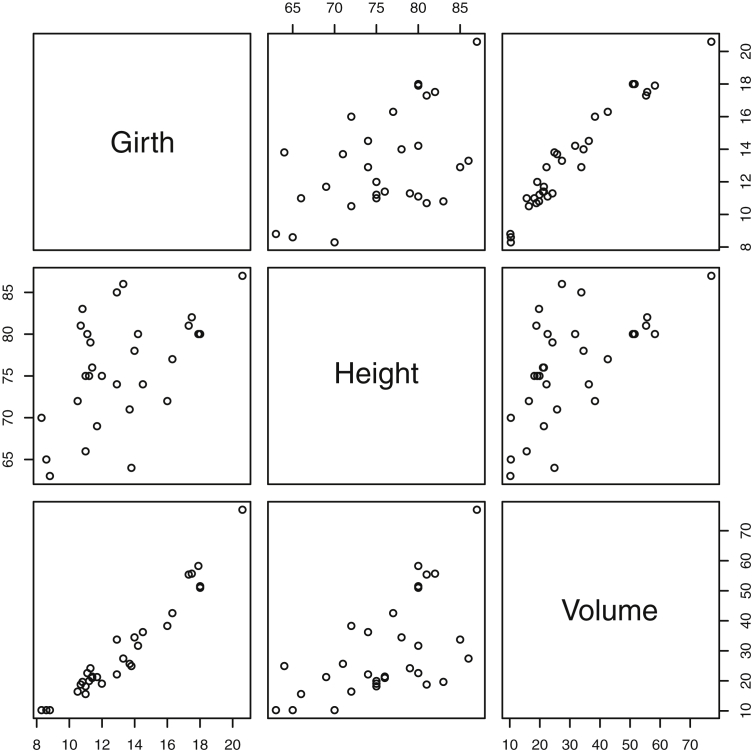
Plot for the *Trees* Data. Girth is measured in inches, while height is in feet, and volume in cubic feet.

**Fig. 8:**
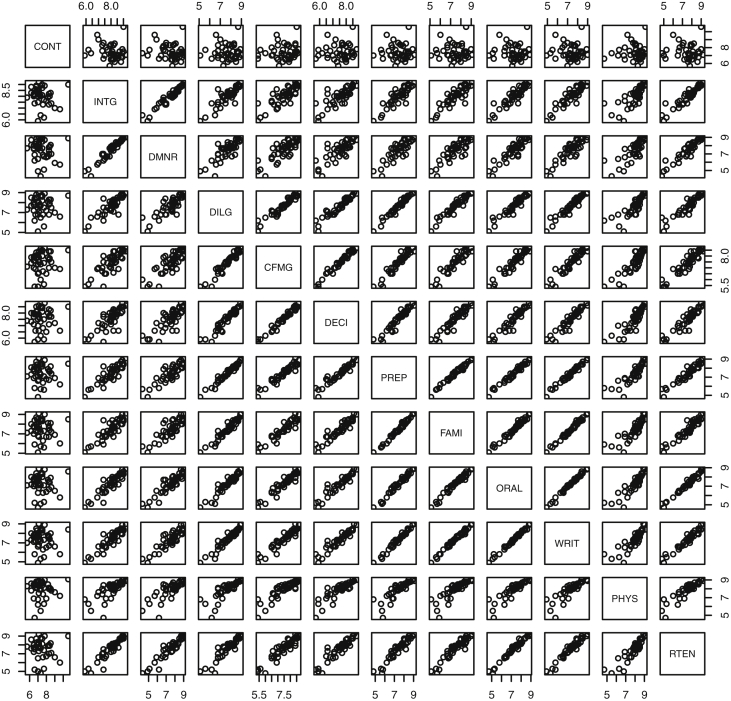
Two dimensional plot for USJudgeRatings data. The plots include pairwise plots of twelve ratings by lawyers for judges from the U.S. Superior Court. Ratings: 1) number of contacts 2) judicial integrity 3) demeanor 4) diligence 5) case flow 6) prompt decisions 7) preparation for trial 8) familiarity with law 9) sound oral rulings 10) sound written rulings 11) physical ability 12) worthiness of retention.

**Fig. 9:**
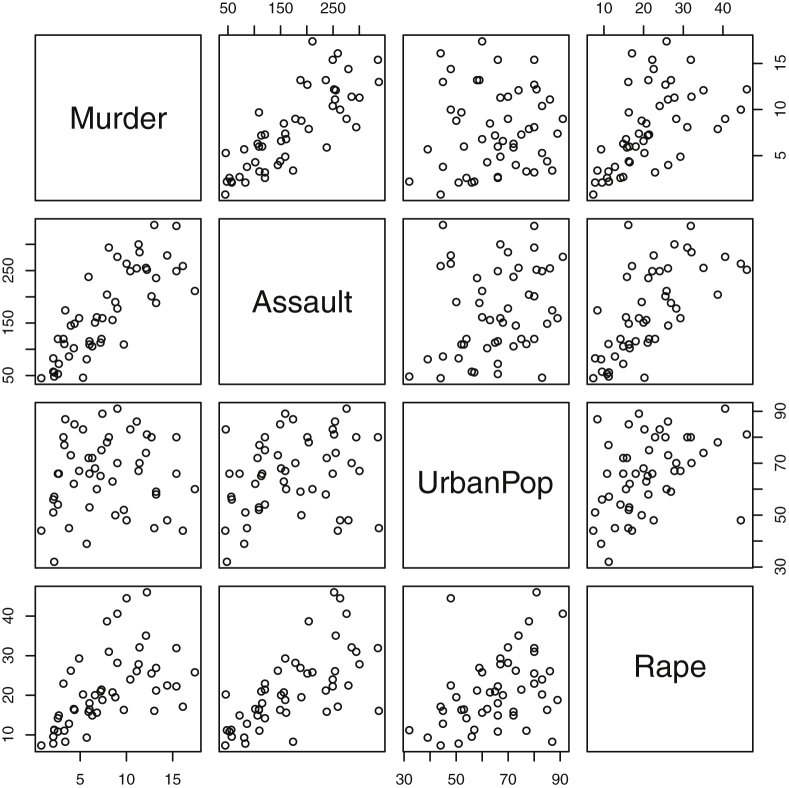
Two-dimensional projections of the USArrests data. Murder, Assault, and Rape refer to the count of arrests per one-hundred thousand residents. Urban population is the proportion of the population within the state living in an urban area.

**Fig. 10:**
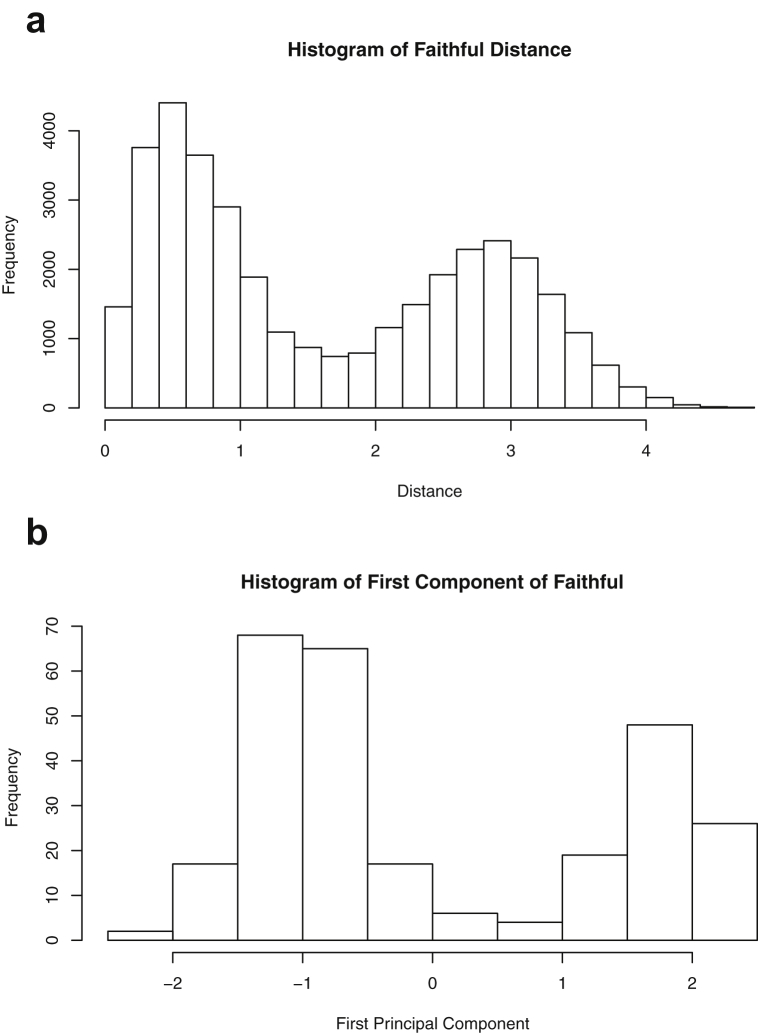
Projections for *Faithful*. The top row (a) includes histograms of the pairwise dissimilarities. The bottom row (b) includes histograms of the first principal component (PCA).

**Fig. 11:**
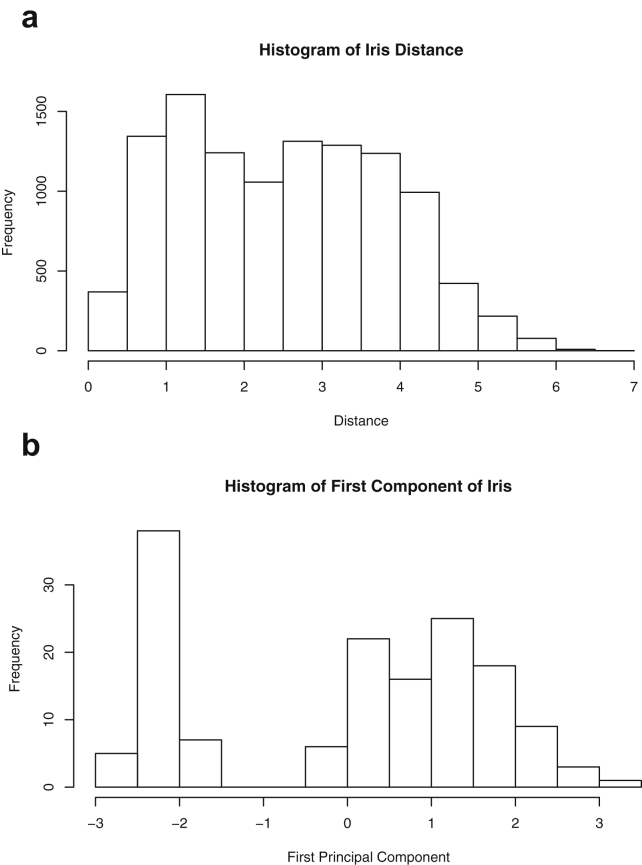
Projections for *Iris*. The top row (a) includes histograms of the pairwise dissimilarities. The bottom row (b) includes histograms of the first principal component (PCA).

**Fig. 12:**
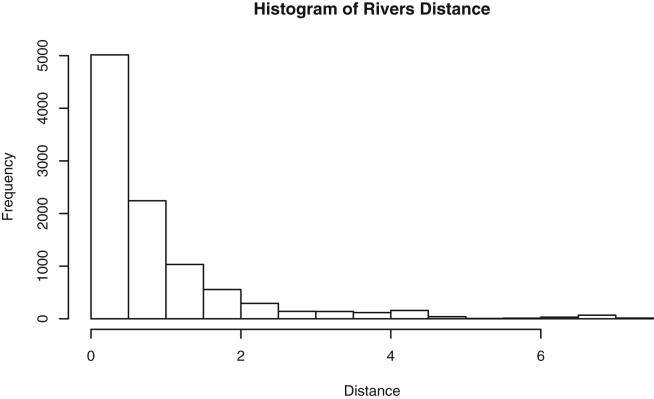
Rivers: Distances.

**Fig. 13:**
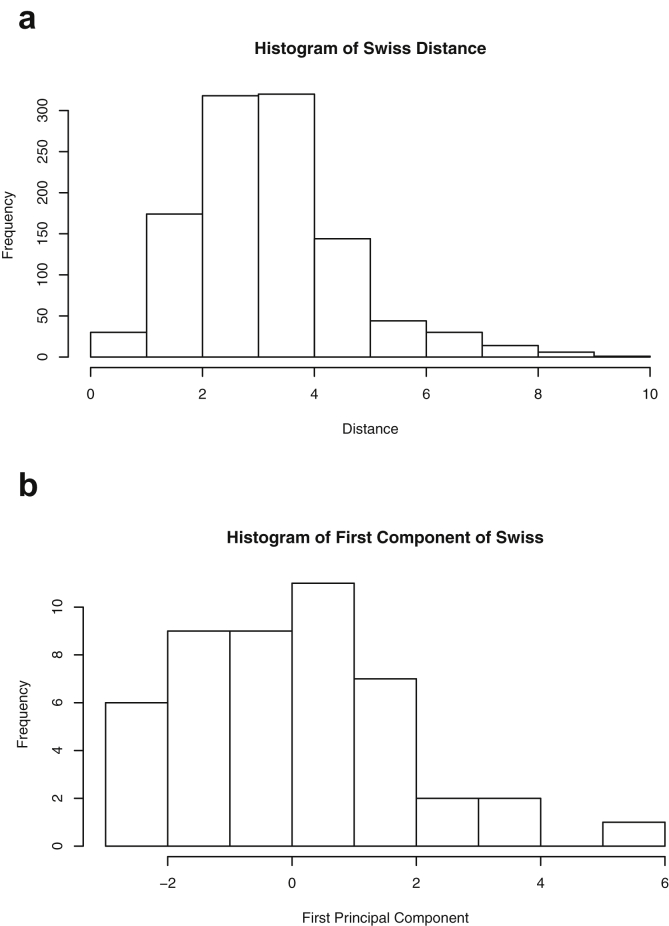
Projections for *Swiss*. The top row (a) includes histograms of the pairwise dissimilarities The bottom row (b) includes histograms of the first principal component (PCA).

**Fig. 14:**
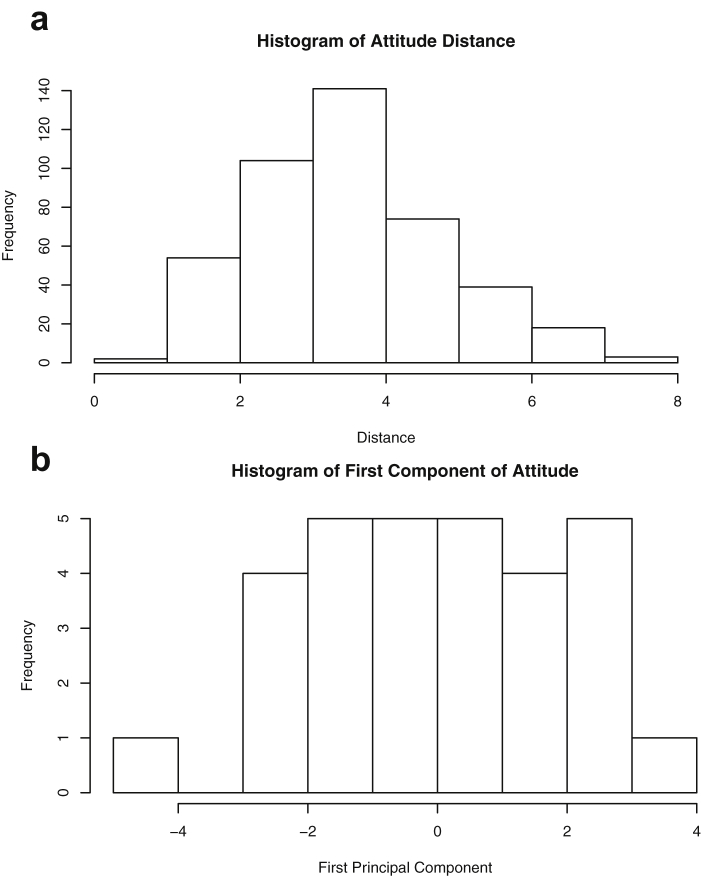
Projections for *Attitude*. The top row (a) includes histograms of the pairwise dissimilarities. The bottom row (b) includes histograms of the first principal component (PCA).

**Fig. 15:**
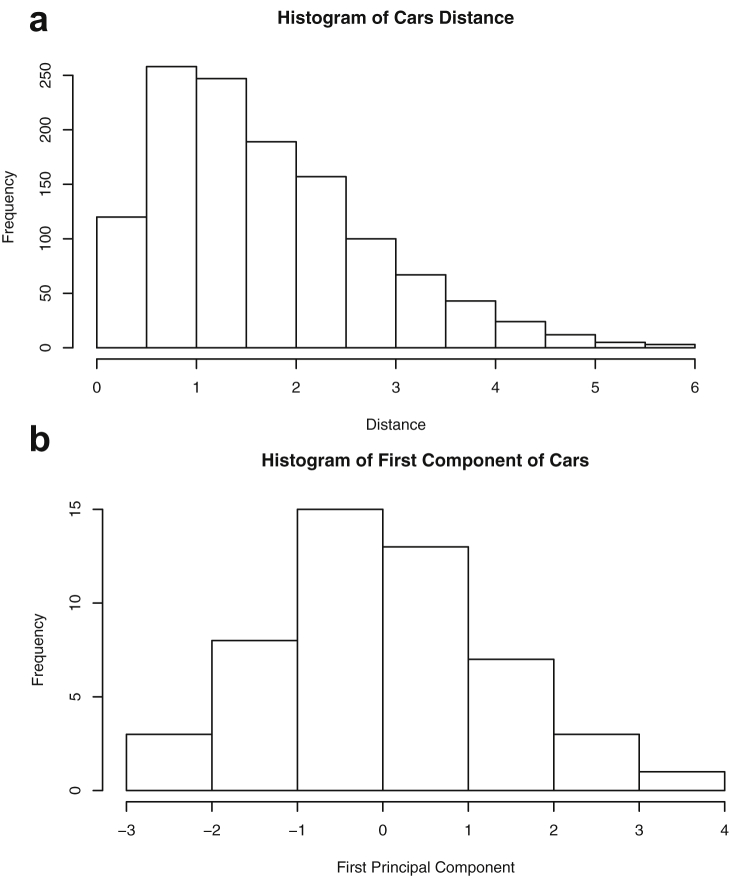
Projections for Cars. The top row (a) includes histograms of the pairwise dissimilarities. The bottom row (b) includes histograms of the first principal component (PCA).

**Fig. 16:**
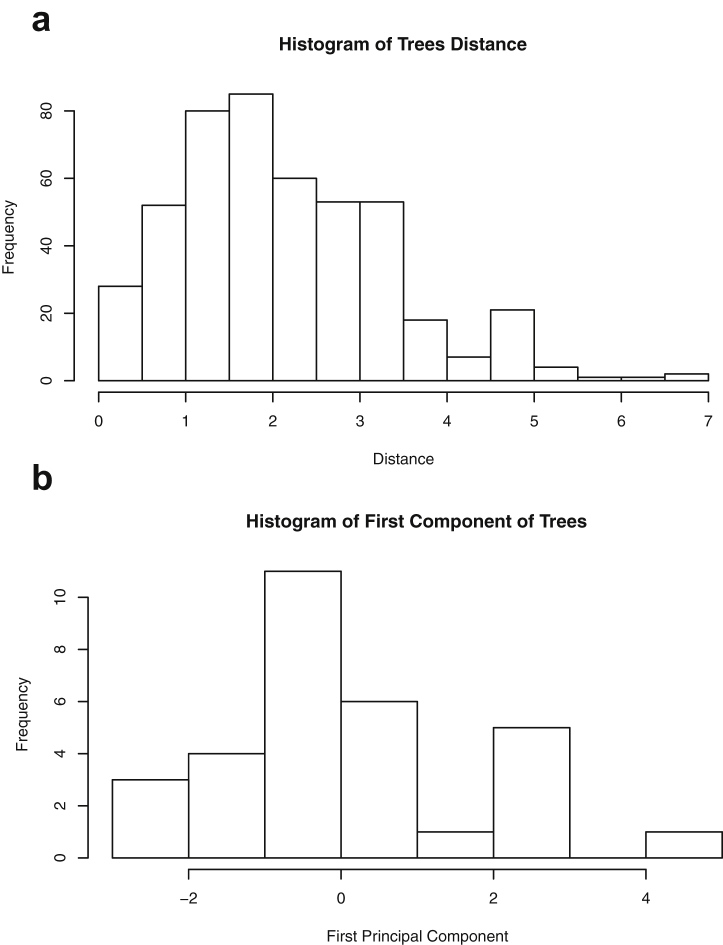
Projections for *Trees*. The top row (a) includes histograms of the pairwise dissimilarities. The bottom row (b) includes histograms of the first principal component (PCA).

**Fig. 17:**
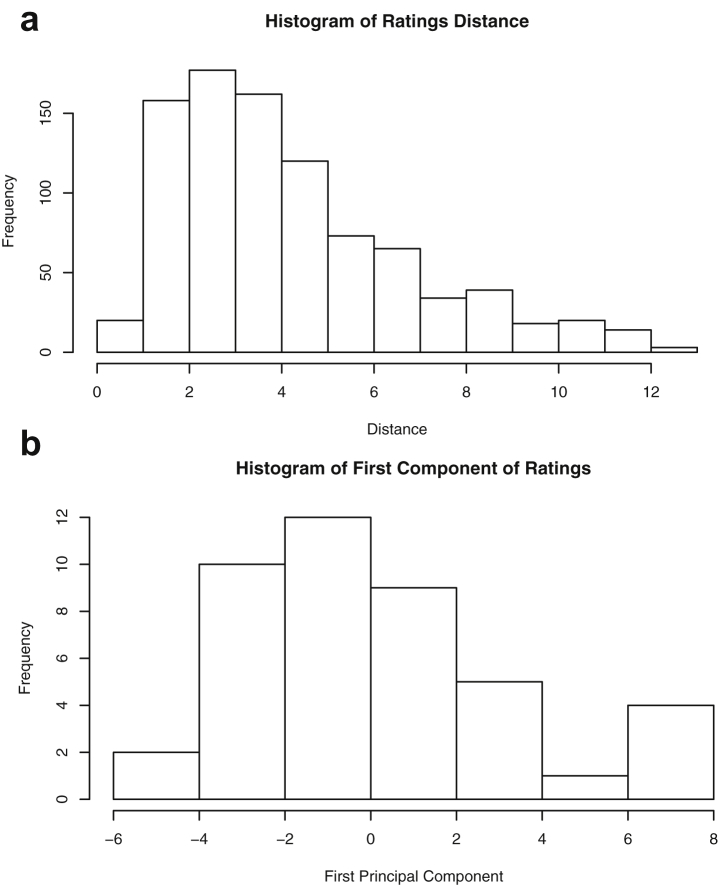
Projections for *USJudgeRatings*. The top row (a) includes histograms of the pairwise dissimilarities. The bottom row (b) includes histograms of the first principal component (PCA).

**Fig. 18:**
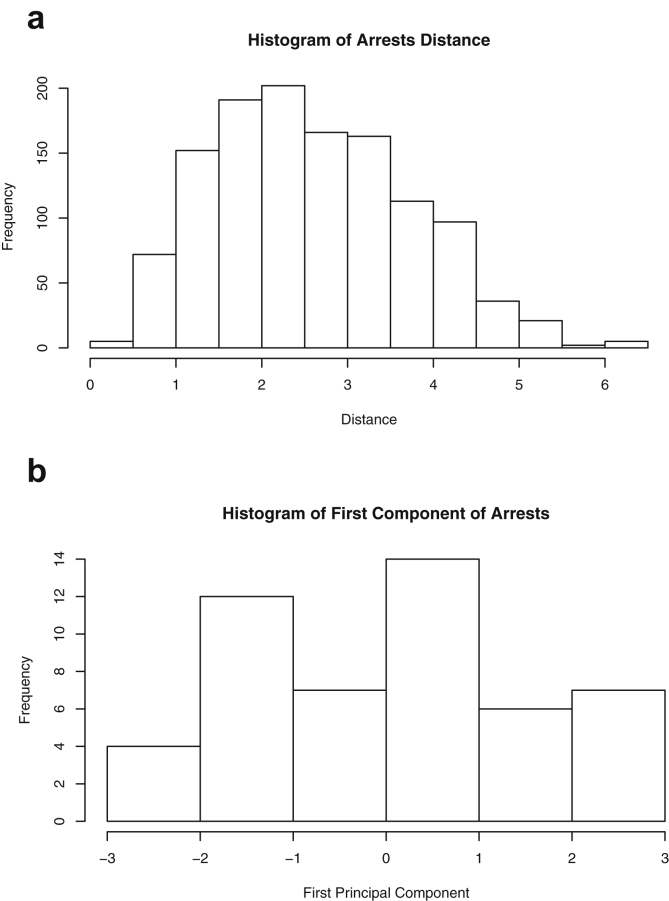
Projections for *USArrests*. The top row (a) includes histograms of the pairwise dissimilarities. The bottom row (b) includes histograms of the first principal component (PCA).

Specifically, waiting and eruption times of Old Faithful are described numerically in Table 1 and plotted in Fig. 1; projections via pairwise distances and PC1 are in Fig. 10. Iris flower measurements are in Fig. 2, distances and PC1 are plotted in Fig. 11, and descriptive statistics are in Table 2. North American river lengths are summarized in Table 3 and plotted in Fig. 3, with pairwise distances in Fig. 12. Table 4 quantifies demographics in Swiss provinces. Pairwise plots of variables, distances between points, and PC1 are in Fig. 4, Fig. 13. Table 5 quantifies employee favorability proportions for seven behaviors, which are plotted in Fig. 5, Fig. 14. Table 6 reports stopping distance and speed for 50 cars. Plots and projections are in Fig. 6, Fig. 15. Table 7 reports tree dimensions, which are plotted in Fig. 7, along with projections in Fig. 16. Table 8 enumerates ratings of US federal judges, plotted in Fig. 8; distances and PC1 are in Fig. 17. Table 9 summarizes state-level crime related variables. Visualizations are in Fig. 9, Fig. 18.

**Table 1:** Descriptive statistics for the *faithful* data. Both variables are measured in minutes. SD and SE, respectively denote the standard deviation and standard error.

	Eruption duration	Waiting time
Min	1.6	43
Max	5.1	96
Range	3.5	53
Median	4.0	76
Mean	3.49	70.90
SD	1.14	13.59
SE	0.07	0.82

**Table 2:** Descriptive statistics for the *iris* data. All variables are measured in centimeters. SD and SE, respectively, denote the standard deviation and standard error.

	Sepal length	Sepal width	Petal length	Petal width
Min	4.3	2.0	1.0	0.1
Max	7.9	4.4	6.9	2.5
Range	3.6	2.4	5.9	2.4
Median	5.80	3.00	4.35	1.30
Mean	5.84	3.06	3.76	1.20
SD	0.83	0.44	1.77	0.76
SE	0.07	0.04	0.14	0.06

**Table 3:** Descriptive statistics for the *rivers* data. SD and SE, respectively, denote the standard deviation and standard error.

	River length (in miles)
Min	135
Max	3710
Range	3575
Median	425
Mean	591.18
SD	493.87
SE	41.59

**Table 4:** Descriptive statistics for the *swiss* data. Fertility is measured via a standardized variable. Agriculture is the percentage of males in the population employed in agriculture. Examination is the percentage of draftees receiving the highest mark on the army examination. Education is the proportion of the population of draftees with education beyond primary school. Catholic is the percentage of the population who identifies as Catholic. SD and SE, respectively, denote the standard deviation and standard error.

	Standardized Fertility	Percent Agriculture	Percent Examination	Percent Education	Percent Catholic	Infant Mortality
Min	35.0	1.2	3	1	2.15	10.8
Max	92.5	89.7	37	53	100.00	26.6
Range	57.5	88.5	34	52	97.85	15.8
Median	70.40	54.10	16.00	8.00	15.14	20.0
Mean	70.14	50.66	16.49	10.98	41.14	19.94
SD	12.49	22.71	7.98	9.62	41.70	2.91
SE	1.82	3.31	1.16	1.40	6.08	0.42

**Table 5:** Descriptive statistics for *attitude* data. Responses correspond to the percentage of favorable responses within a department on the corresponding topic. SD and SE, respectively, denote the standard deviation and standard error.

	Rating	Complaints	Privileges	Learning	Raises	Critical	Advance
Min	40	37	30	34	43	49	25
Max	85	90	83	75	88	92	72
Range	45	53	53	41	45	43	47
Median	65.5	65.0	51.5	56.5	63.5	77.5	41.0
Mean	64.6	66.6	53.1	56.4	64.6	74.8	42.9
SD	12.2	13.3	12.2	11.7	10.4	9.9	10.3
SE	2.2	2.4	2.2	2.1	1.9	1.8	1.9

**Table 6:** Descriptive statistics for the *cars* data. Speed is measured in miles per hour; distance is measured in feet. SD and SE, respectively, denote the standard deviation and standard error.

	Speed	Distance
Min	4	2
Max	25	120
Range	21	118
Median	15	36
Mean	15.40	42.98
SD	5.29	25.77
SE	0.75	3.64

**Table 7:** Descriptive statistics for the *trees* data. Girth is measured in inches, while height is in feet, and volume in cubic feet. SD and SE, respectively, denote the standard deviation and standard error.

	Girth	Height	Volume
Min	8.3	63	10.2
Max	20.6	87	77
Range	12.3	24	66.8
Median	12.9	76.0	24.2
Mean	13.25	76.00	30.17
SD	3.14	6.37	16.44
SE	0.56	1.14	2.95

**Table 8:** Descriptive statistics for the *USJudgeRatings* data. Measurements are ratings by lawyers on judges from the U.S. Superior Court. SD and SE, respectively, denote the standard deviation and standard error. Ratings: 1) number of contacts 2) judicial integrity 3) demeanor 4) diligence 5) case flow 6) prompt decisions,7) preparation for trial 8) familiarity with law 9) sound oral rulings 10) sound written rulings 11) physical ability 12) worthiness of retention.

	1	2	3	4	5	6	7	8	9	10	11	12
Min	5.7	5.9	4.3	5.1	5.4	5.7	4.8	5.1	4.7	4.9	4.7	4.8
Max	10.6	9.2	9.0	9.0	8.7	8.8	9.1	9.1	8.9	9.0	9.1	9.2
Range	4.9	3.3	4.7	3.9	3.3	3.1	4.3	4.0	4.2	4.1	4.4	4.4
Median	7.3	8.1	7.7	7.8	7.6	7.7	7.7	7.6	7.5	7.6	8.1	7.8
Mean	7.4	8.0	7.5	7.7	7.5	7.6	7.5	7.5	7.3	7.4	7.9	7.6
SD	0.94	0.77	1.14	0.90	0.86	0.80	0.95	0.95	1.01	0.96	0.94	1.10
SE	0.14	0.12	0.17	0.14	0.13	0.12	0.15	0.14	0.15	0.15	0.14	0.17

**Table 9:** Descriptive statistics for the *USArrests* data. Murder, Assault, and Rape refer to the count of arrests per one-hundred thousand residents. Urban population is the proportion of the population within the state living in an urban area. SD and SE, respectively, denote the standard deviation and standard error.

	Murder	Assault	Urban population	Rape
Min	0.8	45	32	7.3
Max	17.4	337	91	46.0
Range	16.6	292	59	38.7
Median	7.25	159	66	20.1
Mean	7.79	170.76	65.54	21.23
SD	4.36	83.34	14.47	9.37
SE	0.62	11.79	2.05	1.32

## 2 Experimental design, materials, and methods

Data highlighted in this paper focuses on a subset of nine datasets suitable for cluster analysis: faithful, iris, rivers, swiss, attitude, cars, trees, USJudgeRatings, and USArrests. The following is a brief summary of each dataset; details are in Section 2.1. *Faithful* [2], [7] includes eruption times and waiting times between eruptions of the geyser known as Old Faithful. *Iris* [5] consists of sepal length and width and petal length and width for 150 flowers. *Rivers* [10] provides lengths of 141 rivers in North America. *Swiss* [11] includes six demographic and fertility variables for 47 Swiss provinces. *Attitude* [3] measures worker attitudes for seven items from a survey of employees at a large financial componey. Cars [4] provides speed and stopping distance for 50 cars. Trees [14] includes three measurements each on 31 black cherry trees. USJudgeRatings [8] includes lawyers' ratings of US Superior Court judges. USArrests [10] provides crime variables for each state.

Section 2.1 includes additional details, numerical summaries, and plots for real datasets from the *R datasets* package [12] used in our accompanying study [1]. Section 2.2 focuses on the two unidimensional projections of the data. Code used to produce all items in this paper is included in the file entitled “DIBcode.R.”

### 2.1 Descriptive statistics and raw data plots

Numerical summaries of the data were calculated using the *stat.desc()* function within the *pastecs* package [6]. The summaries we display consist of the minimum, maximum, range, median, mean, standard deviation, and standard error.

Scatter plots for 2-dimensional projections based on all pairs of variables are provided for each dataset. Sets of two dimensional projections are produced using the *plot()* command in *R* [12]. For example, the command *plot(iris)* produced the projections in Fig. 2.

The following subsections provide background on each dataset used and the variables contained therein.

#### 2.1.1 Faithful

The *faithful* dataset [2], [7], contains two variables for the Old Faithful geyser. The first is the eruption duration, and the second is the waiting time between eruptions. Both are measured in minutes. Fig. 1 displays the data. Table 1 summarizes the statistical properties of these features.

#### 2.1.2 Iris

The *Iris* [5] dataset is well-documented and consists of 150 recorded flower measurements, spanning across 3 species of 50 measurements each. The studied flowers are members of the following three species: iris setosa, versicolor and virginica. The variables, all measured in centimeters, include the sepal length and width and petal length and width. Descriptive statistics for the four features are included in Table 2. These features, along with the species, are displayed in Fig. 2.

#### 2.1.3 Rivers

In the *rivers* [10] dataset, the length, in miles, is recorded for 141 major rivers in North America. The mean, median, extrema, standard deviation and standard error of the river lengths are provided in Table 3. The data contains only one variable. Therefore, it does not have a two-dimensional projection. Instead see the one-dimensional plot in Fig. 3.

#### 2.1.4 Swiss

The *swiss* [11] data includes 47 French-speaking nineteenth-century Swiss provinces, each of which contains six measures of socio-economic status and fertility. Fertility is measured using a standardized variable [13]. The remaining five variables are percentages correspond-ing to agricultural workers, high scores on the army exam, education past primary school, members of the Catholic religion, and infant deaths. Pairwise plots of the 47 points for each pair of measures are included in Fig. 4. Numerical summaries are found in Table 4.

#### 2.1.5 Attitude

The dataset *attitude* [3] consists of seven employment behavior variables measured based on a survey completed by employees within a large company in the financial sector. Thirty departments were randomly selected, and the approximately thirty-five employees within which were aggregated to calculate the seven measures. The responses represent the proportion of favorable responses within each department to each of seven questions.

The seven questions could have favorable or unfavorable answer to the following themes: overall rating, handling of employee complaints, the department does allow special privileges to some individuals and not others, the company presents ample opportunity to learn, raises are given based on performance, evaluations are critical, and employees consider that there are opportunities for advancement. Descriptive statistics and plots of the raw data are found in Table 5 and Fig. 5.

#### 2.1.6 Cars

Recorded in the 1920s, *cars* [4] consists of 50 observations and two variables, representing speed and stopping distance. Speed is measured in miles per hour. Stopping distance is measured in feet. Table 6 includes numerical summaries of the stopping distance and speed for each of fifty cars. Fig. 6 contains a plot of these two features.

#### 2.1.7 Trees

The *trees* [14] dataset is depicted in Fig. 7. Features include measurements of the girth, height and volume of timber in 31 felled black cherry trees. The units for girth, height, and volume are inches, feet, and cubic feet. Descriptive statistics on these variables are included in Table 7.

#### 2.1.8 USA judge ratings

The dataset *USJudgeRatings* [8] contains 43 observations with ratings from lawyers on twelve elements related to judges from the U.S. Superior Court.

The following are the twelve elements: number of contacts of lawyer with judge, judi-cial integrity, demeanor, diligence, case flow managing, prompt decisions, preparation for trial, familiarity with law, sound oral rulings, sound written rulings, physical ability, and worthiness of retention.

Descriptive statistics are found in Table 8. Plots of scores for the forty-three judges from each pair of lawyers are included in Fig. 8.

#### 2.1.9 USA arrests

The dataset *USArrests* [10], depicted in Table 9 and Fig. 9, contains measurements from 1973 for each of the fifty states on 4 variables: urban population percentage and the number of arrests per 100,000 residents for assault, murder, and rape.

### 2.2 One-dimensional projections

One-dimensional summaries of all data are discussed in this section. Two projections are examined side by side. The first is the set of pairwise distances between the points. The second is the first component extracted from a principal component analysis. Both summaries of the projected distributions are shown using histograms. For more background on these projections and examples, please see the accompanying article [1].

Histograms were made using the *hist()* function in *R*. For histograms of the set of dis-similarities, the distance metric employed in the present manuscript is Euclidean distance, defined as the square root of the sum of the squares of the differences between the values of each variable for a pair of observations. Distances were computed using *dist()* function in *R*. All data was scaled to have unit variance before analysis using the *scale()* function in R. Principal component analysis [9] was executed in *R* via singular value decomposition using the *prcomp()* function. The first principal component of each scaled dataset was extracted and examined visually with histograms.

The distributions of the pairwise Euclidean distances and first principal component are found in side by side plots, shown in Fig. 10, Fig. 11, Fig. 12, Fig. 13, Fig. 14, Fig. 15, Fig. 16, Fig. 17, Fig. 18. Code to produce the plots is included in the supplementary material. Because this paper is not focused on classification, the species variable from the *iris* data is not used in dimension reduction. Rather, the unidimensional reductions are computed based only on the first four features. Histograms of the pairwise distances and first principal component for *iris* are found in Fig. 11. For all other datasets, all variables were used for.

Dimension reduction. Pairwise distances for the *rivers* data are included in Fig. 12. However, no dimension reduction by principal component analysis is executed, because the data is already only one-dimensional and principal component analysis is not recommended for such data.
