# Supplementary material for: Descriptive statistics and visualization of data from the R datasets package with implications for clusterability
Source: Data Brief. 2019 May 24;25:104004. doi: 10.1016/j.dib.2019.104004 (PMC6612012; doi:10.1016/j.dib.2019.104004)
Supplement: Multimedia component 1 [file mmc1.docx]

Conflict of Interest Statement

We do not have any conflicts of interest for this manuscript.
